# Nondestructive Inspection of Water Pipes: A Review

**DOI:** 10.3390/s26061994

**Published:** 2026-03-23

**Authors:** Rileigh Nowroski, Piervincenzo Rizzo, Liam Byrne, Adeline Ziegler

**Affiliations:** Department of Civil and Environmental Engineering, University of Pittsburgh, 3700 O’Hara Street, 742 Benedum Hall, Pittsburgh, PA 15261, USA; ren28@pitt.edu (R.N.); ljb100@pitt.edu (L.B.); abz20@pitt.edu (A.Z.)

**Keywords:** pipe inspection, nondestructive evaluation, structural health monitoring, optical methods, stress waves, review article

## Abstract

Pipe networks assure the transportation of primary commodities such as water, oil, and natural gas. Quantitative and early detection of defects avoids costly consequences. Due to low cost of water, high-profile accidents, and economic downturns, the research and development of nondestructive evaluation (NDE) and structural health monitoring (SHM) technologies for freshwater mains and urban water networks have received less attention with respect to the gas and oil industries. Moreover, the technical challenges associated with the practical deployment of monitoring systems and the fact that most water pipelines are buried underground demand synergistic interaction across several disciplines, which may limit the transition from laboratory to real structures. This paper reviews the most prominent NDE/SHM technologies for freshwater pipes. The challenges that said infrastructures pose, as well as the methodologies that can be translated into SHM approaches, are highlighted. The scope of this review is to provide a holistic view of the physical principles, the success, and the technological challenges associated with the inspection and monitoring of freshwater pipelines.

## 1. Introduction

The structural integrity of engineering systems such as pipelines, railroads, bridges, and offshore platforms, is essential to prevent failures that may have catastrophic consequences. Structures may experience unexpected manmade or natural harsh events that yield to long-term deterioration. In addition, as the global population continues to grow and the tonnage of commodities to be transported increases across the globe, the need for effective nondestructive evaluation (NDE) and structural health monitoring (SHM) strategies to ensure optimal performance of engineering infrastructures becomes more critical.

NDE and SHM aim to assess the condition of existing infrastructure and detect damage at early stages, thereby extending service life and preventing failures. NDE is typically conducted periodically by certified personnel using commercially available technologies. However, periodic inspections can do little when flaws develop or become critical between scheduled maintenance intervals. To address this inherent limitation, the implementation of cost-effective SHM methods is on the rise. SHM enables continuous, non-invasive assessment of structural conditions by shifting the maintenance paradigm from “time-based” NDE, in which structures are inspected at fixed intervals, to “permanent-based” monitoring. In this framework, one or more sensors continuously monitor the structure and stream time-series data to processing units that flag, locate, and characterize damage as it occurs [1,2].

Water pipelines serve fundamental societal needs, particularly in regions facing water scarcity and limited economic development [3]. In densely populated areas, leakage problems can lead to pipe bursts, ground collapse, and other accidents, affecting life and operational safety [4]. In the U.S., the drinking water infrastructure comprises more than 3.2 million kilometers of underground pipes operated by nearly 150,000 public water systems [5]. Some of the oldest pipes were laid in the 19th century, while those installed post-World War II have average lifespans of 75 to 100 years, meaning that even relatively newer segments are reaching or have exceeded their design life but are still awaiting replacement due to inadequate funding [6]. As of 2023, the average life expectancy of these pipes stands at just over 78 years, representing a 6-year decrease since 2018 [5]. These systems remain physically and figuratively out of sight, yet are subjected to various stresses, including increasing rainfall, that lead to costly failures [7]. Recent statistics [8] echoed by ASCE [5] estimate that ~126 B m^3^ of water is lost annually, resulting in more than $187 B in lost revenue.

The U.S. Environmental Protection Agency estimates that water collection systems have a total replacement value between $1 and $2 trillion [9]. As these networks age and populations grow, both the risk and impact of failures increase correspondingly. The ASCE’s 2025 Report Card for America’s Infrastructure assigned grades of C- for drinking water, a score that has not changed from the 2021 report. The estimated funding gaps from 2024 to 2033 total $309 billion for drinking water and $690 billion for stormwater and wastewater systems combined. According to [6], approximately 260,000 water main breaks occur annually in the U.S., resulting in roughly $2.6 billion per year in maintenance and repair costs. With an average failed water main age of 53 years and 33% of all water mains exceeding 50 years old, an estimated 452,000 miles of pipes have surpassed their useful service lives. Yet only 69.9% of utilities maintain pipe-replacement programs, and merely 44% conduct regular condition assessments [6]. Water loss from leakage averages 11% across the U.S. and Canada, with leaks often remaining undetected until significant damage or economic loss has occurred [6,10]. Nonetheless, there are clear signs of progress in the U.S. In the timeframe 2018–2023, a 20% annual reduction in water main breaks has been reported, most of which resulted from a nearly 8% decrease in the use of cast iron and asbestos cement pipes that are responsible for the highest rates of breakage across all pipe materials [6] and replaced by ductile iron and polyvinyl chloride (PVC). Similar situations have been reported elsewhere.

In the UK, the length of water pipes (mains) owned by water companies is equal to 351,387 km, and an estimation of 3 million liters of water leaked each day on average during the April 2021–March 2024 timeframe [11]. While statistics from China could not be found, it was reported that leakage detection technology in large and medium-sized Chinese cities is mainly based on manual listening, which is considered to have low reliability, poor interference resistance, and requires a lot of manpower with low detection efficiency [4]. In South Korea, water supply pipelines operational since the 1960s are buried underground and exceed 4000 km, more than 50% of the entire network [12,13]. In Australia, there are over 120,000 km of drinking water pipelines [14]. On a larger scale, there are about 4.3 million km of pipes in Europe [15], and leakages in such large networks are almost inevitable. According to [16], water leakages around the world range from 5% to as high as 70% of the total water production.

The complexity of water pipe management extends beyond funding constraints and the capillarity of the water distribution networks. Underground pipes are inherently difficult to inspect, let alone monitor, and no single method suffices for comprehensive evaluation, especially after reactive approaches are adopted. The Grand Canyon’s main water line exemplifies the consequences of reactive maintenance and the challenges of buried pipe inspection. Completed in 1970 with a designed 30-year lifespan, this 12.5-mile pipeline has experienced over 85 breaks since 2010, with each repair costing approximately $25,000—not including lost revenue from turned-away visitors due to closures [17]. This case underscores a broader trend: aging pipe infrastructure is deteriorating and failing faster than NDE and SHM research and implementation can progress. Additional complexity to water transmission and distribution infrastructure includes internal geometries, connections, inspection chambers, hydrants, valves, and pumps whose locations may be uncertain. Moreover, pipe materials vary across different installation periods and generations, with the condition of older infrastructure often unknown [9]. Effective condition assessment requires multiple complementary methods deployed strategically. However, the vast scale of existing networks and the labor requirements of current inspection techniques create a pressing need for proactive, automated, and unmanned monitoring approaches.

The scope of this review article is to provide a comprehensive background of the NDE methods proposed or currently implemented in practice to inspect or monitor water pipes. Research and development in pipeline inspection have been predominantly focused on oil and gas infrastructure, driven by high-profile accidents, economic incentives, and the strategic importance of hydrocarbon resources. Consequently, freshwater systems have received comparatively less attention due to water’s lower economic value and the technical challenges inherent in monitoring and inspecting buried pipes. This paper provides guidance for researchers developing novel solutions or improving existing methodologies to address the inspection and monitoring challenges facing freshwater infrastructure. Building upon an earlier review by one of the authors [18], we present a state-of-the-art update on research, development, and commercialization spanning the period from 2015 to 2025. While some authors cluster inspection and leak detection technologies in in-pipe detection, on-pipe contact detection, and ground-based non-contact detection [4], we examine methodologies across four broad categories: visual inspection, stress wave-based methods, electromagnetic (EM) methods, and probabilistic approaches. For each category, we discuss technological evolution, challenges, advantages and limitations, applications in both commercial and academic settings, and future research directions. To this end, the recent advancements of the Internet of Things (IoT), artificial intelligence (AI), and robotics have stimulated great progress in automated damage detection and classification, as well as in pipe management [3,19].

The review was conducted by using two databases beginning in September 2025: Google Scholar and Scopus. The foundational query “nondestructive evaluation” AND “water pipes” was launched, and the results were narrowed by including individual keywords such as “guided ultrasonic waves”, “visual inspection”, “acoustic leak detector”, etc. Inclusion criteria were peer-reviewed articles and technical reports submitted to funding agencies. Primary exclusion criteria were patents and conference abstracts. Secondary exclusion criteria were short communications, conference papers, and websites, for which, however, a few exceptions were made in the absence of other documentation. Other exclusion criteria were methodologies related to remote sensing, such as unmanned aerial vehicles, infrared-based camera use, GPS, etc. In the absence of other documentation, pamphlets from manufacturers and service providers were considered. It is noted here that some review papers recently published targeted specific areas of interest within the large umbrella of pipe inspection/monitoring. For example, Tran et al. [20] reviewed the inspection methods for PVC mains and their suitability for being integrated into continuous monitoring systems. They categorized the methods into five groups: (1) sound wave; (2) fiber optic sensing; (3) hydraulic monitoring; (4) multiple discrete sensors; and (5) other methods. Abdelhafidh et al. [21] and Ali and Choi [22] surveyed a wireless sensor network pipeline monitoring system without any specific clustering.

**Figure 1:**
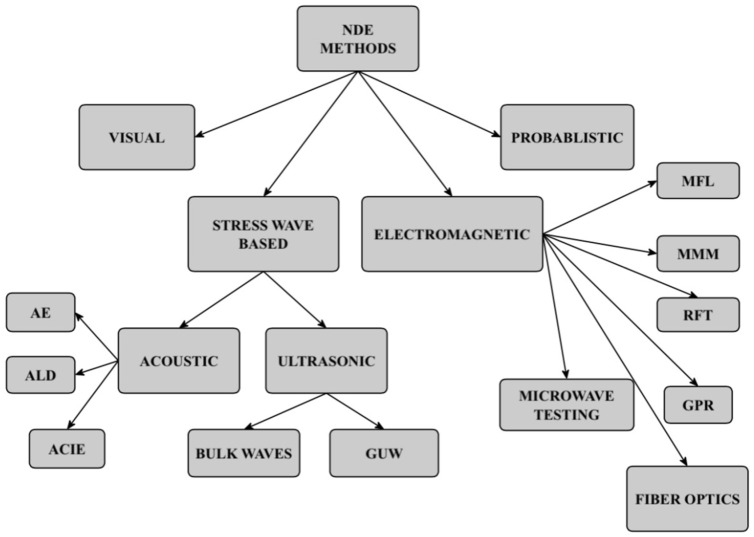
Overview and grouping of methodology reviewed. Flow of methodology from general to more specific categories is shown via arrows.

The paper is organized as follows. Section 2 delves into visual inspection. Section 3 covers all the methods based on the propagation and detection of stress waves. The section was subdivided into the passive methods of acoustic emission (AE) and acoustic leak detection (ALD), and the active methods related to the excitation, propagation, and detection of bulk and guided ultrasonic waves (GUWs). Section 4 covers the broader range of methods based on electromagnetic testing, including the emerging applications of fiber-optic sensors. Section 5 discusses the probabilistic approaches that aid in the management and prioritization of intervention. Finally, Section 6 ends the paper with some concluding remarks. Figure 1 visualizes the methods discussed here.

## 2. Visual Inspection

Visual-based NDE is often the first and most basic form of NDE, valued for its simplicity, cost-effectiveness, and ability to quickly identify obvious problems. It can be performed with the naked eye or enhanced using tools like magnifying glasses, borescopes, cameras, or advanced imaging technologies, while being implementable in any environment with sufficient lighting. All visual inspection methods are prone to subjectivity in damage assessment and labor-intensive implementation. When applied to pipes, other limitations include the inability to comprehensively cover the vast networks of buried assets and the inability to detect problems that have not emerged on the surface.

Overall, industrial inspection of freshwater pipes (and sewer lines) tends to be manually operated using tethered systems mounted with cameras and closed-circuit television (CCTV), where the footage is manually searched for damage and blockages [23,24]. One of the simplest tools is the RIDGID^®^ See-Snake^®^ Standard Pipe Inspection System [25] that consists of a camera reel attached to either a 61 m or 99 m push cable, reinforced with fiberglass and flexible enough to travel through sharp 90° bends, yet stiff enough to push the camera head long distances. The device is ideal for inspecting 50 to 300 mm lines.

**Figure 2:**
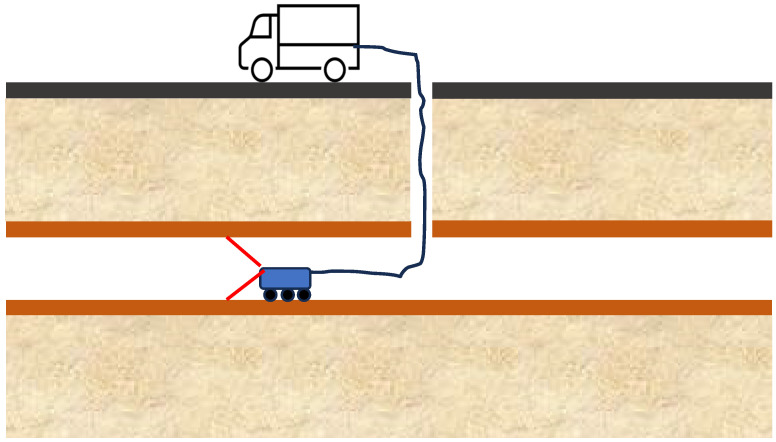
Schematic of in-pipe visual testing with CCTV. Pipe boundaries are represented by the thick orange lines, field view represented by thin red lines. Images are typically recorded onsite and then replayed and post-processed in-house.

Camera-aided visual inspection technologies can be categorized based on the use of CCTV, typically mounted on remote-controlled crawlers (Figure 2), QuickView systems, and manhole zoom cameras. Each of these methods requires personnel to review footage and control either the crawler or the zoom camera. These techniques also require adequate lighting conditions and mandate that pipes be cleaned of sludge and debris to enable accurate surface condition assessment [26,27].

CCTV is the first technology applied to corrosion detection [28] and has been widely used since the 1980s. Any CCTV-based system is mainly composed of a control platform, a crawler, a camera, a cable tray, and a recorder. Vision information recording the defect conditions, including rust layer, scaling, corrosion, perforation, and cracks within the pipeline, can be accessed by controlling the crawler carrying a high-frequency camera [28]. Technology is more common in sewer lines than in freshwater mains. These images or videos are translated by experienced engineers or image recognition software into information usable for pipeline condition ratings, according to standards issued by the authorities [27,28,29]. When deployed along sewer lines, CCTV is obviously unable to detect areas covered with sewage and sludge. A technical limitation of traditional CCTV-based methods is the lack of depth perception inherent to single-lens camera systems, which prevents comprehensive pipe characterization. Localizing defects discovered can prove difficult when using CCTV, zoom cameras, or QuickView techniques, even when image quality is acceptable, which cannot be guaranteed, particularly with QuickView approaches [30].

To address depth perception limitations, Zhang et al. [31] proposed panoramic cameras to create three-dimensional (3D) reconstructions of pipe interiors. This technique identifies identical feature points across multiple panoramic images, which are then compiled into a unified 3D model. This process also enables correction of the fisheye distortion inherent to 360-degree cameras. This method requires operator-controlled equipment deployment and manual reconstruction of pipe geometry from collected footage. To overcome the limitations related to human expertise and labor, as well as to prevent results from continuing to be subject to evaluator bias and experience level, machine vision systems were proposed to automate the analysis of water and wastewater pipe video and have been under active development. Automating footage evaluation eliminates the need for personnel to manually review hours of video and reduces subjectivity through AI-based fault detection, resulting in faster assessment times and improved resource efficiency [30].

Similar to CCTV technology, Quickview is mainly composed of an intelligent display controller, transmission cable, camera, telescopic pole, and U-probe. It is highly integrated, battery-powered, compact, and lightweight, suitable for field or mobile operating conditions. The disadvantage is that it is time-consuming, and the detection distance is limited [27,28]. Manhole zoom camera consists of photographing the inner part of a pipeline in a range of about 20–30 m, using the camera installed at the manhole by adjusting the sight distance [27,30,32].

Overall, traditional vision-based camera-based inspection systems require the examination of large collections of videos. This is time-consuming and labor-intensive. In addition, the interpretations are biased by the operator’s work experience [28,29]. With recent developments in artificial intelligence and image recognition, the efficiency and accuracy of these vision detection techniques have been greatly improved [33]. Michael et al. [19] applied deep learning architectures such as You Only Look Once version 8 (YOLOv8) to train an AI to differentiate between intact and damaged pipe conditions by identifying recurring patterns associated with specific defect types. While human involvement remains necessary for data collection, footage uploading, and results verification, YOLOv8 models have demonstrated mean average precision up to 90% in individual experiments and 74.7% across multiple experimental trials. This performance substantially reduces the volume of footage requiring direct human inspection, showing significant promise for scaling visual inspection efforts [19].

Rayhana et al. [30] combined visual and non-visual methods to enhance accuracy and defect localization capabilities. Their multi-modal framework integrates five different non-invasive methods, namely impact-echo testing for structural integrity assessment, acoustic and ultrasonic methods for non-visual surface evaluation, eddy current testing for material characterization, visual inspection for direct surface assessment, and ground-penetrating radar for bedding material evaluation. The idea behind this holistic approach is to enable comprehensive characterization of defect depth, location, severity, and pipe specifications. Each method’s limitations are compensated for by the strengths of others, providing more robust and reliable condition assessment than any single technique alone.

Advances in mobile robotics have presented an opportunity to replace these manual inspections with continuous observation of pipe networks and to solve the challenges posed by confined space inspection, offering enhanced efficiency, accuracy, and safety [34]. Popular industrial robotic devices for pipe inspection include PipeDiver^®^ [35], Sahara^®^ [36], and SmartBall^®^ [37]. These technologies will be discussed in the next section as they are based on the detection of stress waves, and the cameras, when mounted, represent a complementary tool for inspection. However, according to Aitken et al. [38], none of the current mobile robot technologies can autonomously monitor/inspect pipes over a large spatial scale and longtime durations.

## 3. Stress Wave-Based Methods

### 3.1. Acoustic Methods

Acoustic methods exploit the propagation of low-frequency mechanical waves (below 10 kHz) through either the pipe, the water, or the terrain surrounding the asset [26]. Acoustic methods can be divided into two large groups: Acoustic Emissions (AE) and Acoustic Leak Detection (ALD).

AE is formally defined as the ‘‘release of transient elastic waves produced by a rapid redistribution of stress in a material” [39]. AE is a very popular NDE method to characterize structural behavior and detect the onset of structural problems in a wide variety of laboratory settings [40,41] and active infrastructures [42]. AE can also be considered as the first SHM paradigm, especially when applied to structures such as bridges [43,44]. When applied to pipe monitoring, AE-based strategies utilize an array of fixed sensors installed on the pipes of interest to detect transient waves originating from a change within the material, which is usually permanent and irreversible, such as the growth of a crack. For example, the AE method is suitable to detect damage onset and/or propagation but cannot detect existing or “silent” damage. In PCCP and reinforced concrete pipes, AE enables the detection of steel reinforcement breakage, crack onset, and propagation within the concrete. Other sources of emissions are friction, crack growth, turbulence, leaks, and corrosion.

When a pressurized pipeline’s liquid leaks, an acoustic continuous signal is generated by frictional vibration with air, silt, or when the liquid is ejected from the pipe [4]. This signal propagates along the pipeline, along the liquid commodity, and along the medium surrounding the asset. This sound can be picked up using in-pipe (where sensors attached to a device flow through the pipe network) or on-pipe systems. Regardless, the sensing approach is typically labeled ALD. On-pipe implementation may use a listening stick in contact with the pipe, a noise recorder, and a correlation meter [10,45]. Devices that can be stuck into or on the ground without contact with the pipe are said to be non-contact [4,46]. A recent example of such an application is found in [4], where a ground-based acoustic array detection technology for leakage detection in buried water supply pipelines was proposed. The technology is based on built-in piezoelectric acceleration sensors and software to define the initial time, duration, number of acquisition times, interval time, and final time. Above-ground noise analyzers can also be considered as non-contact devices, although they are listening devices attached to pipeline fittings, such as valves or fire hydrants, operating below 100 Hz [20]. Anguaiano et al. [47] conducted a comprehensive survey test with different commercial equipment of noise correlation leak detection, such as Gutermann ZoneScan Alpha, Echologics LeakFinderRT, and SebaKMT Correlux, using 33 m PVC and 27 m ductile iron pipes. They found that Correlux yielded the best results, detecting leaks from 4 to 30 L per minute, and accurately pinpointed their location to within 1 m as well as the leak rate, with 100% detection and 0% false positives. However, when tested in the field with multiple leaks and sensor distances of up to 15 m, the method’s reliability was reduced to less than 20% [20].

AE and ALD differ because the former detects transient waves originating from material failures, and it usually uses passive detectors such as accelerometers or piezoelectric transducers attached to the pipe. The latter detects the continuous sound caused by a leak, and the signal is detected by microphones often inserted in the water or on the ground surrounding the asset. Both AE and ALD offer several advantages: they are non-invasive, can interrogate large sections of pipeline, detect internal defects, accommodate various pipe materials, and enable failure prediction before catastrophic events occur. However, acoustic sensors are predominantly deployed and operated manually, making inspections expensive, time-consuming, labor-intensive, and susceptible to human error. Additionally, environmental noise can interfere with the measurements and compromise the proper interpretation of the results as can sensor characteristics such as thickness and adhesive properties [48]. Recent developments in autonomous robotics have reinvigorated research into acoustic methods, enabling more sensitive and accurate NDE approaches [9]. The emergence of integrated systems of “intelligent” technologies used by water utilities to monitor, control, and optimize water distribution and treatment in real-time, often called Smart Water Networks (SWNs), has further advanced the field by incorporating permanent acoustic sensors and IoT connectivity into SHM frameworks, building upon established ultrasonic and passive/active acoustic sensing technologies [48,49]. Sensors include accelerometers and hydrophones to enable continuous monitoring of buried pipes and provide enhanced sensitivity to small and emerging defects. When integrated within SWN architectures where data are continuously logged, these systems can identify anomalous recordings indicative of developing failures.

Both methods can be implemented reactively or proactively. In the first case, sensors are deployed after leaks are suspected or failures occur. ALD typically uses portable acoustic correlators and listening devices. However, smaller cracks and incipient defects often escape detection by portable sensors due to elastic-wave dissipation and environmental noise interference. Furthermore, implementation remains labor-intensive and reactive, relying on failures to persist or worsen to enable detection [49].

A notable implementation was conducted by the University of Adelaide in South Australia, where 23 high-speed sensors—including acoustic sensors—were installed across Adelaide City. These sensors, magnetically attached to pipe fittings within fire hydrants, continuously record data that are analyzed to identify trends and normal operational patterns, which are then compared against hydraulic model predictions. Significantly, this study revealed that theoretical models of the water system could not accurately predict regular flow volumes or event impacts, demonstrating that theoretical models alone are insufficient and must be supplemented with empirical data from physical systems. As the authors noted, “controlled testing and/or high-density transient data logging (temporary) is required to better understand the physical influences affecting varying transient propagation in different parts of the network” [50]. This finding underscores the challenges inherent in probabilistic approaches discussed in Section 2, particularly regarding uncertainty in aging infrastructure, where material properties and installation configurations are poorly documented.

The primary challenge in implementing these systems is the substantial environmental and non-leak acoustic interference, particularly in densely populated urban areas, which can generate false positives or mask small defects, resulting in false negatives. Optimal performance requires careful sensitivity calibration through normalization of expected non-leak vibration sources and implementation of threshold-based alerting that triggers only upon persistent increases in leak characterization values [49]. Importantly, continuous monitoring has proven unnecessary; comparable accuracy can be achieved through once-daily recording and data upload [10].

A technology commercialized by Mueller’s Echologics division includes the EchoWave Acoustic Water Leak Detector, which focuses on proactive leak detection using signal processing algorithms designed to filter environmental noise. An example of field deployment of this technology was presented by [51] and included the cities of Ontario (Canada), where over 60 miles of large-diameter pipes were inspected, and the United Kingdom. For the latter, the system identified leaks totaling 5.5 ML/day of water loss. Additional Mueller acoustic products include:

- LeakFinder-ST System: Deployed for over two years on asbestos cement mains in Gold Coast, Australia. Released in 2014, this system automatically estimates acoustic velocity in water pipes, improving leak localization accuracy. Enhanced electronics provide superior performance for quiet leaks, while simplified interfaces enable broader user accessibility [52].
- ePulse Condition Assessment Technology: Validation studies by New Jersey American Water in 2014 confirmed that acoustic testing results matched findings from physical pipe examinations [53].
- EchoShore-TX System: Successfully detected a large leak in a 42-inch water main responsible for approximately 100,000 gallons per day of water loss [54].

These commercial systems demonstrate effective environmental noise reduction and precise leak detection capabilities, supporting proactive and continuous monitoring of water distribution networks [55].

**Figure 3:**
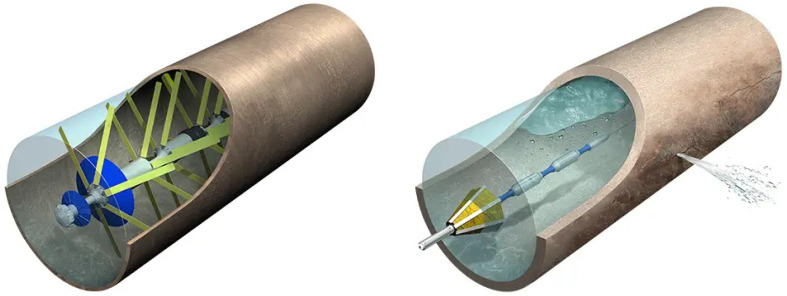
Scheme of the Pure Technologies PipeDiver^®^ (**left**) (Figure retrieved from [35]) and Sahara^®^ (**right**) (Figure retrieved from [36]).

WRC [56] describes three notable commercial systems that combine robotics and ALD: PipeDiver^®^ (Figure 3), Sahara^®^ (Figure 3), and SmartBall^®^. Sahara^®^ is a tethered inspection tool used for pinpointing leaks and carrying out pipeline condition assessment in real time while the pipeline is in service. The tool can be used in potable water trunk mains, raw water mains, wastewater rising mains, and sewers. According to the manufacturer [36], the tool can detect pinhole leaks using passive acoustic sensor technology and can capture the sound of trapped air, which can adversely affect pipeline flow and magnify the effects of transient pressure events. Other capabilities include confirming pipeline alignment, providing an internal visual inspection, and capturing live videos. The latter is guaranteed by a CCTV camera that displays real-time footage of the pipeline interior. The Sahara^®^ platform is designed for pressurized water pipelines 150 mm and larger. During operation, the platform can locate the pipeline with sub-meter accuracy, providing global positioning system (GPS) coordinates for points of interest. Since 1997, utilities around the world have relied on the Sahara platform to inspect more than 7050 miles (11,350 km) of pipeline and detect over 8820 leaks [36].

Finally, SmartBall^®^ is a free-flowing (rolling) inspection tool that can be deployed for long distances, while the pipeline remains in service, to detect leaks and air pockets and map water and wastewater pipelines. The system can cover long inspections in a single deployment, and it can be actively tracked throughout the entire inspection. Owing to the spherical geometry, the tool can be easily deployed through existing features and hydrants and can navigate tees and vertical sections of pipe even under low-flow conditions. Similar to Sahara^®^, SmartBall^®^ is equipped with an acoustic sensor able to detect pinhole-sized leaks and to recognize the sound of trapped gas. This is achieved within 1.8 m. According to Xylem [37], the SmartBall^®^ platform has inspected more than 10,600 miles (17,050 km) of pipeline and detected over 4775 leaks since 2005.

An advancement of the free-swimming in-pipe spherical detector (SD) was proposed by Jian et al. [57] with the purpose of overcoming the vulnerability of the system to collision noise interference, which increases the difficulty of leak identification. Jian et al. [57] proposed an automatic identification method by incorporating an audio spectrogram transformer into deep learning. The method builds upon the construction of a pipeline leak voice dataset using real pipeline acoustic signals captured by the SD and related to normal rolling, collision, and leak. Then, the dataset is augmented by combining data augmentation and transfer learning. Finally, the AST model is applied to pipeline leak sound identification and leak point localization. The experiments conducted to prove the method showed that this method can accurately identify and locate 1 mm aperture leaks, and the localization errors under 1 MPa and 0.5 MPa conditions are 2.46 m and 3.07 m, with relative errors of 0.37% and 0.47%, respectively.

Recently, Wang et al. [4] proposed a novel ground-based acoustic array detection technology for leakage detection in buried water supply pipelines. The array on the ground is intended to capture the acoustic signals originating from underground leaks. Ad hoc signal processing based on spatial interpolation was proposed to reconstruct the leakage sound field. In addition, a formula for estimating the breakage size by using the calculated sound intensity value was fitted using the field experimental data to assist the detection work. The methodology is at the development stage, and a single field test was reported.

### 3.2. Air-Coupled Impact Echo (IE)

Impact-echo (IE) testing primarily targets concrete structures. IE involves mechanically striking the surface of a structure with a small impactor, e.g., an instrumented hammer or a stainless-steel ball, to generate low-frequency stress waves that travel through the material to be inspected. These waves reflect off internal flaws, voids, delamination, or backwalls, and return to the surface where they are detected by one or more contact or non-contact sensors placed near the impact point. By analyzing the frequency content of the reflected waves in the time or frequency domain, engineers can determine the thickness of structural elements, locate internal defects, and assess the integrity of the material. In engineering practice, IE is applied for evaluating concrete slabs, bridge decks, tunnel linings, and other plate-like structures, offering a relatively quick and portable method for detecting subsurface anomalies that are invisible to visual inspection [58,59,60,61].

For water pipe inspection, IE is typically employed to detect defects within concrete pipe walls. However, research has also investigated IE’s capability to identify voids around concrete sewage pipelines, which may indicate leakage or pose infrastructure risks by contributing to sinkhole formation or ground collapse that further damages pipes. Results demonstrate that IE alone provides unreliable void detection [62]. As frequently observed in NDE, IE performs optimally when paired with complementary methods or when implemented using innovative data-collection approaches. Given the often poor quality of in-service concrete pipes, air-coupled impact echo (ACIE) has been developed specifically for testing buried concrete infrastructure [63]. ACIE employs air-coupled sensors (such as microphones) rather than contact sensors, distinguishing it from standard IE. Research demonstrates that ACIE satisfactorily detects thickness loss, delamination, and soil voids around pipes—capabilities that conventional IE failed to achieve reliably.

ACIE requires three instrumental components: an impactor, a receiving transducer (such as a microphone), and a data acquisition system (DAQ), employed analogously to standard IE methodology. The impactor generates stress waves in the pipe, the transducer detects the reflected waves, and the DAQ performs numerical modeling of recorded signals to produce interpretable waveforms. Implementation of noise suppressors has been proposed to mitigate environmental noise interference and improve signal-to-noise ratios [63]. Among the techniques reviewed in this article, IE testing is perhaps the least implemented technology in industry practice.

### 3.3. Ultrasonic Testing: Bulk Waves

Market-size, UT is the most common NDE method [64]. UT uses stress waves in the ultrasonic (above 20 kHz) range to detect internal flaws, characterize material properties (e.g., Young’s modulus), and infer geometric characteristics (e.g., thickness) [65,66]. UT includes many different techniques depending upon the way ultrasounds are generated and detected, the position of the transducers (or other elements) used to transmit and detect the wave, the direction of propagation of the waves, and the algorithms used to process the signals.

**Figure 4:**
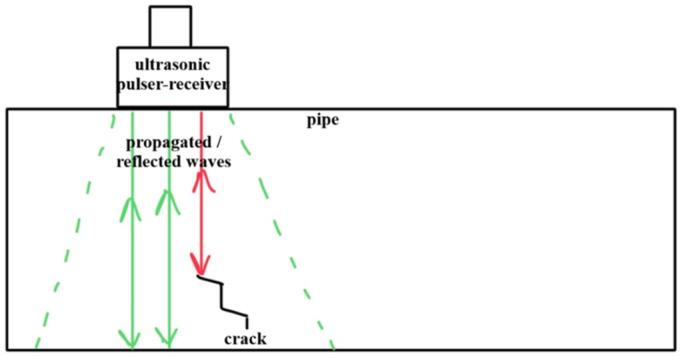
Bulk UT propagation and detection of stress waves. Green lines represent normal propagated and reflected waves; red lines represent waves that have encountered a fault and will result in an abnormal reading. Dashed lines represent the inspection field.

A conventional bulk UT system integrates a pulser–receiver unit that generates high-voltage electrical pulses with a transducer. The transducer converts electrical energy into ultrasonic acoustic energy, which propagates as a wave through the material. When the propagating wave encounters a discontinuity—such as a crack, void, or internal flaw—a portion of the energy reflects back (Figure 4). The transducer receives this reflected signal, converts it to an electrical signal, and transmits it to a display device. The resulting trace typically displays reflected signal amplitude versus time-of-flight (elapsed time from pulse generation to echo reception). Signal travel time correlates directly with propagation distance, enabling inspectors to characterize material properties and locate defects or material loss.

UT can inspect pipes both internally and externally; however, external inspection presents challenges as pipes may require excavation and surface preparation—impractical for buried water and wastewater infrastructure. Despite limitations in detection area coverage and relatively high costs and time requirements, bulk wave UT remains valuable for accurately quantifying defects and continues to be relevant for targeted spot testing in field applications and research developments [67]. The practical relevance of bulk-wave UT for buried pipe inspection was demonstrated by Zhu et al. [68], who developed and validated an ultrasonic bulk-wave approach for condition assessment of buried plastic water pipes. Their study focused on identifying void formation and loss of ground support in high-density polyethylene (HDPE) and polyvinyl chloride (PVC) pipes. Using a water-coupled transducer with a 10 MHz center frequency, the researchers successfully detected machined grooves and slots measuring 1–2 mm in 6 mm-thick PVC plates. The method also proved capable of identifying major cracks in PVC and detecting voids in both PVC and HDPE materials, demonstrating the feasibility of high-frequency bulk-wave UT for plastic pipe inspection under controlled conditions [68].

Advancements in bulk-wave UT are driven not only by hardware improvements but also by sophisticated signal processing techniques. Researchers in Norway addressed a common challenge in internal pipe inspection: degradation of image resolution when the distance between in-pipe sensors and pipe walls varies due to diameter changes, internal obstacles, or imperfect robotic alignment [69]. To overcome this limitation, they proposed a Synthetic Aperture Focusing Technique (SAFT) incorporating a Cylindrical Phase Shift Migration (CPSM) algorithm. This approach mathematically refocuses ultrasonic data post-acquisition, effectively compensating for sensor misalignment and variable standoff distances. The study demonstrated that applying CPSM focusing on scans acquired outside optimal inspection zones produced reconstructed topography maps with 96% correlation to ideally focused reference scans [69]. These results indicate that advanced post-processing can enhance the high-resolution imaging range of ultrasonic sensors, enabling reliable detection of pitting and corrosion even under non-ideal inspection conditions. Overall, these studies illustrate that while conventional bulk-wave UT faces challenges related to inspection coverage and signal attenuation, continued innovation in transducer design, deployment strategies, and signal processing substantially enhances its applicability. Such developments are critical for improving the practicality of bulk-wave UT in inspection and long-term monitoring of aging water and wastewater pipeline systems.

PipeDiver^®^ is a free-swimming, high-resolution pipe inspection tool. It does not require service interruption and can inspect the condition of 40 km or more in a single deployment. Inserted and extracted under pressure through a 0.3 m tapping, PipeDiver^®^ can be used in pipe diameters from 0.3 m–3 m. Using water flow for propulsion, navigating bends with fins, this robotic system uses ultrasonic (for metal) or electromagnetic (for concrete) sensors to detect pipe wall damage, sending data to identify corrosion, broken wires, or wall loss without service interruption. This inspection technology provides high-resolution pipe-wall analysis and enables remaining life assessment for metallic and PCCP mains, as well as wire-break detection for PCCP. Broken wire wraps, sedimentation, corrosion, and anomalies can be detected with this system. Onboard cameras can also spot cracks, debris, and other issues [35].

Examples of implementations of PipeDiver^®^ are found in Jappy et al. [70], who reported the results of the condition assessment of 18.63 km PCCP on the 1650 mm Branch II Aqueduct in Winnipeg, Manitoba. Owing to the nature of the pipe, electromagnetic inspection technology was used to locate and identify pipes that have broken prestressing wire wraps. The presence of electromagnetic anomalies was deemed consistent with prestressing wire damage, ranging from 5 to 20 broken wire wraps. Another example of PipeDiver^®^ technology with ultrasonic testing was presented by Bernal Chimal et al. [71] who reported the condition assessment of a 2.9 km long 800 mm riveted steel transmission main section in the City of Vancouver (Canada) conducted in 2019 with the scope of identifying areas of wall thickness loss and assessing lining and out-of-roundness. Five pipe sections with wall-loss anomalies were found, one of which was validated one year later when the City of Vancouver excavated one of the pipe sections that showed wall-loss anomalies.

Finally, a through-thickness UT system mounted on a robot was presented by Wickramanayake et al. [14] for the inspection of spray-lined pipes. Ultrasonic pulses centered at 5 MHz were delivered to the pipe via a custom-designed coupling device and were tested in the lab and in a 0.6 m diameter out-of-service water pipeline in Sydney (Australia).

### 3.4. Guided Ultrasonic Waves

Guided ultrasonic waves (GUWs), also known as long-range UT (LRUT), are ultrasonic waves propagating along a medium where at least one dimension is much smaller than the other two. Examples of such media are plate-like structures, including shells, rods, pipes, and rails. For pipes, the waves travel down the length of the pipe, being reflected by flaws in the pipeline and detected by the sensor that monitors the entire circumference. Unlike conventional ultrasonic bulk waves that propagate through the volume of a material, GUWs travel along specific paths defined by the geometry and boundary conditions of the structure, with their energy confined within the waveguide. These waves can propagate over considerable distances, making them effective for inspecting/monitoring large areas. The main limitation of GUWs is that they are multimodal and dispersive, which means that different modes travel at different velocities depending on frequency and geometry. While these characteristics can provide rich information about the health of the structure being inspected, they require careful mode selection and signal interpretation [65,66].

Owing to the characteristics of GUWs and the possibility to use them both for inspection and continuous monitoring, there has been an explosion of research and development over the last 20 years on the use of pipe inspection and monitoring using GUWs (see, for example, the review of Ghavamian et al. [72] and Olisa et al. [73]). While seminal works have been presented in many scientific papers and different research groups, including a few from the US [66,74,75,76,77] and the UK [78,79,80,81], many investigations are being carried out everywhere to enhance knowledge and improve sensitivity and monitoring range [82,83,84,85]. Some of these works have been translated into commercial products, mostly targeting oil and gas pipelines to be inspected from the outer walls.

Marketwise, many companies offer hardware, software, and services for GUW-based inspection and monitoring of pipes. While the primary application remains in the oil and gas industry, the commercial systems can be applied to water mains and distribution networks. Teletest Focus+, commercialized by Eddyfi Technologies, is one of the earliest examples. It comprises rings which contain 24 transmitters and 24 receivers operating in the 10–300 kHz frequency range. The rings are held by a pressurized collar that can be rapidly installed around the pipe circumference without requiring surface preparation or coupling agents, as they utilize dry-coupled technology. The transducers are designed and arranged to simultaneously operate longitudinal and torsional waves. The transducers are allotted in three-ring modules and spaced at 30 mm and 45 mm [86]. The Teletest Focus+ system is integrated into proprietary software that controls signal transmission, detection, and processing. The inspection/monitoring performance targets various forms of metal loss, including general corrosion, pitting, and erosion.

**Figure 5:**
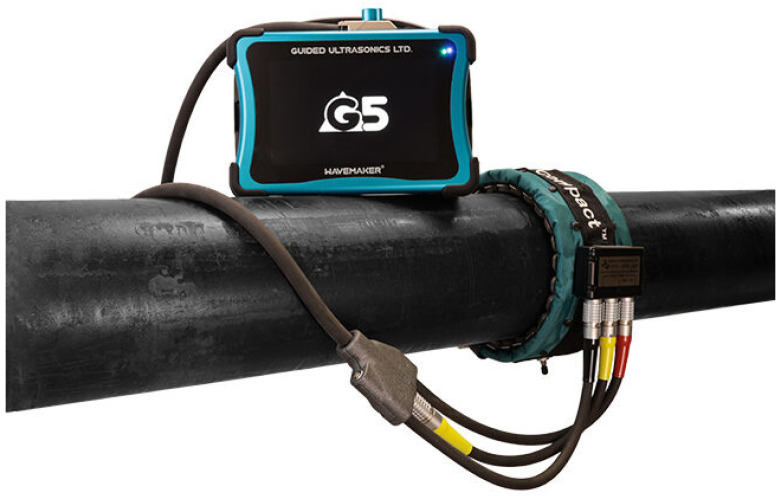
Photo of the Wavemaker^®^ G5 by Guided Ultrasonics LTD (Brentford, UK). Photo retrieved from Ref. [89].

GUL Wavemaker^®^ [87,88] (Figure 5), commercialized by Guided Ultrasonics LTD., leverages two decades of research at the Imperial College (London, UK) [78,79,80,81] and continuous industry development. The unit used a collar made with an array of piezoelectric transducers to generate and detect specific wave modes (including the fundamental torsion mode) and specialized software for signal processing and data interpretation. GUL Wavemaker^®^ is globally recognized as a reliable system for pipes under different physical conditions and environments.

Guided Wave Analysis LLC [90] is another long-range GUW-based inspection system that uses the principles of magnetostriction to generate and detect guided waves. The system stems from research and developments at the Southwest Research Institute in Texas [91,92] and has been tested by others in lab applications such as Panda et al. [93].

Another service provider and proprietary commercial system is represented by Guidedwave [94], which offers a suite of comprehensive NDE/SHM solutions for pipe inspection/monitoring. The solutions vary in terms of transduction mechanisms (magnetostriction, piezoelectricity), probes (phased-array, single-channel/multiplexed, and handheld scanning hardware platforms specifically designed to operate our guided-wave probes), and signal processing. This company was founded by the author of seminal books and scientific papers in the area of GUWs [66,74] and patents (e.g., Borigo et al. [95], Rose et al. [96]).

Despite the existence of many commercial players, GUW-based testing for pipes remains an active research area. Research focuses on improving the design of the transducers and enhancing signal-processing capabilities. For example, Huang et al. [82] designed a GUW-based NDE system based on an adaptive wavelet threshold denoising algorithm to enhance the signal-to-noise ratio of echo signals. However, the proposed advancements are rarely tested in water-filled pipes, let alone existing urban or rural assets. Despite these advances, some challenges still exist, including the inherently dispersive and multimodal nature of GUWs, complex scattering behavior associated with irregular defects, and (when applied as an SHM strategy) baseline signal variations caused by changing environmental [85] and operational conditions.

## 4. Electromagnetic Methods

### 4.1. Magnetic Flux Leakage

**Figure 6:**
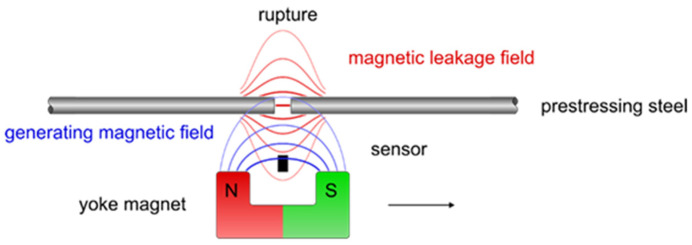
Visualization of MFL. Photo retrieved from [98].

Magnetic flux leakage sensing (MFL) is an electromagnetic-based NDE method for detecting surface-breaking or near-surface defects in ferromagnetic materials. MFL testing relies on the distortion of magnetic field lines when a defect interrupts the normal flow of magnetic flux through a magnetized ferromagnetic component. When a strong magnetic field is applied to steel or other ferromagnetic materials using permanent magnets or electromagnets, the magnetic flux tends to follow the path of least magnetic reluctance (resistance), which is typically through the material itself. However, when a defect is present, some of the magnetic flux leaks out of the material and into the surrounding air, and becomes detectable by electromagnetic sensor arrays, such as Hall sensors, magnetoresistive elements, or induction coils, positioned near the test surface [97] (Figure 6).

MFL-based inspection systems are particularly advantageous for large-area screening applications. When applied to pipe inspection, MFL techniques can be classified into two main categories: single-sensor and multi-sensor [99,100,101]. In single-sensor systems, defects are characterized in a one-dimensional domain by establishing relationships between defect features and MFL signal characteristics. In the former, image processing is used to determine defect location, orientation, and quantity, and signal processing techniques are used to estimate defect width [99]. The two main methods for this, model-based and model-free methods, both have their respective downsides. Model-based methods work by applying physical models to the assumed defects, and the difference between simulation values and original measurements is compared. Then, the assumed defect size is modified iteratively to approach the real size. This has the downside of being time-consuming and difficult for complex systems. The model-free method mainly relies on the establishment of a mapping relationship between the features of measurements and the defect size by data fitting or a training method of networks, ignoring the underlying physical process. This often involves the use of statistical modeling, Fourier transformations, and wavelet transformations [100].

Commercial tools for pipe inspection are commonly known as intelligent PIGS (Pipeline Inspection Gauges), which utilize MFL technology to inspect thousands of kilometers of oil and gas pipelines from the interior, traveling with product flow while continuously assessing wall thickness and detecting corrosion or mechanical damage. PIGS can travel remotely through the pipe and are equipped with permanent magnets or electromagnets to magnetize the surrounding pipe wall [97]. When applied to pipe inspection, the method has several strengths: it does not require removal of coatings or surface contaminants, operates effectively at high inspection speeds, is moved by the flow of the commodity, and provides good sensitivity to volumetric metal loss. Limitations include reduced sensitivity to narrow cracks oriented parallel to the magnetization direction, limited penetration depth (typically effective for defects within approximately 80% of wall thickness), and applicability restricted to ferromagnetic materials. Due to the nature of the inspection method, it is useful for buried or inaccessible pipes, which applies to many water and sewer lines. MFL requires adequate surface-to-sensor contact, and as such is only applicable for cleaned and unlined pipes [97].

Recent developments in MFL-based inspection include the use of Visual Transformation Convolutional Neural Networks (VT-CNN) to improve the interpretation of single-sensor MFL data [100]. The visual transformation layer can transform the original MFL measurements to a three-dimensional (3-D) image at any angle of view. Though faster, there is inherent inaccuracy due to correlation and interpretation being foundational principles. To overcome this issue, the researchers created a VT-CNN. These transformed images can better describe defects by transforming the original MFL measurements into a 3D image from any angle of view. Through the comparison experiment, it was proven that this method can improve the estimated accuracy of length by 26.9%, width by 27.1%, and depth by 33.3%, which can reduce the error by up to 4.6 mm in length, 5.9 mm in width, and 7.2% in depth [100]. This has yet to be widely commercialized due to the necessity of having a massive data pool from which the VT-CNN system can be trained, but it does show incredible potential for accurately sizing defects in the future when it is cost-effective enough for use in the water industry [100].

In comparison, multi-sensor MFL systems build on the limitations of single sensor approaches by collecting data from several sensors at once, which gives a clearer picture of how the magnetic field is distributed around the pipe [99,101]. By combining these measurements through data fusion techniques, researchers can improve the accuracy of defect detection and characterization. The fused data is often processed using machine learning methods, which help identify patterns in the magnetic field and make predictions about defect type and severity. However, a major downside of multi-sensor MFL is that many status quo data fusion methods, like wavelet analysis or k-nearest neighbor, are not well-suited to analyze multi-sensor data because they cannot account for the nonlinear relationships between multi-sensor sources [99,101].

To address this, a new multi-sensor fusion framework was developed to enhance magnetic flux leakage (MFL) data and improve defect characterization when sensor information is incomplete. The approach introduces a space-vector-based fusion method that combines both the amplitude and direction of the magnetic field, allowing for a more accurate representation of its spatial distribution [101]. In addition, improved conditional generative adversarial networks (icGANs) were used to reconstruct and enhance multi-sensor data by refining the generator loss function, resulting in higher-quality MFL information for more reliable defect evaluation. In a case study, for example, icGANs have the lowest depth estimation error as compared to cosine and k-nearest neighbor methods [101].

Jeon et al. [12] developed an in-pipe inspection robot system designed for large-diameter (0.9–1.2 m) water pipes with an MFL sensor module. The robot consisted of the front and rear driving parts, with the inspection module located centrally, and was powered by 22 motors, including eight wheels with motors positioned at both the bottom and the top for propulsion. To ensure that the robot’s center aligns with that of the pipeline during operation, lifting units were incorporated. The inspection system included cameras and LiDAR sensors at the front and rear to monitor the internal environment of the pipeline. The ability to perform the inspection was validated through driving experiments on a field test bed about 1 km long.

### 4.2. Metal Magnetic Memory (MMM)

The Metal Magnetic Memory (MMM) method, also known as the Magnetic Memory Testing (MMT) technique, is a passive electromagnetic NDE approach that detects stress concentration zones and early-stage damage in ferromagnetic structures. The method is based on the occurrence of magnetic domain reorientation under mechanical stress in ferromagnetic materials. When a ferromagnetic component is subjected to mechanical loading in the presence of Earth’s weak magnetic field or residual magnetization from manufacturing processes, irreversible changes occur in the magnetic domain structure at locations of maximum stress concentration. These stress-induced magnetic anomalies persist even after the load is removed. The MMM inspection involves scanning a sensitive magnetometer or magnetic field sensor, typically a Hall sensor or fluxgate magnetometer, across the test object’s surface without requiring prior magnetization or surface preparation. The measured parameter is generally the normal component of the magnetic field or its gradient, which is plotted as a function of position along the scan line. Stress concentration zones, incipient cracks, material discontinuities, or regions of microstructural degradation are purportedly indicated by characteristic anomalies in the magnetic field distribution. These physical principles make the method feasible for preventative pipeline monitoring [102].

When water and wastewater pipelines experience loads from operation, ground movement, or residual manufacturing stresses, these forces cause permanent changes in the orientation of magnetic domains at areas of high stress. By detecting these changes, MMM can identify regions that are more likely to develop defects in the future. The method has the advantage of being noncontact and lightweight due to its passive nature [102]. MMM only works for ferromagnetic materials, and it is still in the developmental stage. Most existing studies have been carried out in controlled laboratory settings, which do not accurately reflect real-life operating conditions [103]. In addition, interactions between nearby structural components can interfere with magnetic measurements during field inspections, affecting the reliability of the results. As a result, developing magnetic sensors that are more resistant to external interference is a critical area for future research [103]. Gong et al. [104] developed a high-sensitivity MMM inspection system that uses an embedded Linux real-time platform for efficient data processing. The system applies magnetic anomaly characterization algorithms that show high accuracy in identifying stress concentration zones in pipelines and is designed to be portable with automated stress recognition. It outputs a stress concentration severity index, F, which quantitatively represents the level of stress concentration in the pipe. The system was validated through fatigue testing and X-ray diffraction (XRD) analysis, demonstrating its ability to accurately identify plastic deformation zones and provide early warnings up to 3200 load cycles before failure, corresponding to approximately 98% of the fatigue life. In addition, the system is relatively low-cost and largely operator-independent, making it well-suited for proactive pipeline integrity monitoring. While MMM adoption in water pipeline applications has been limited by challenges related to data processing complexity, as discussed earlier, pattern interpretation, and overall system cost, the results of this study suggest that the proposed system has strong potential to overcome these barriers.

MMM is primarily relegated to research and lab tests at present, but some commercial applications have been seen recently, with promising results. Energodiagnostika, based in Russia, developed a non-invasive magnetic inspection system for gas and oil pipelines buried at depths of two meters or more. The system allows an operator to walk along the pipeline route at speeds of at least 2 kmh while continuously collecting magnetic data. Using this approach, pipeline segments experiencing high stress and increased susceptibility to damage can be identified. These critical sections are then targeted for more detailed follow-up inspections to locate and characterize specific defects [102]. This study shows promising results, with clear stress concentration zones being visible through visually identifying areas with extreme spikes in data very close together.

### 4.3. Remote Field Testing (RFT)

**Figure 7:**
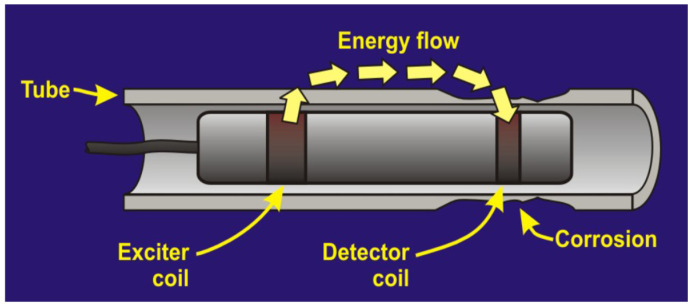
Schematics of the RFT method. Photo retrieved from [105].

**Figure 8:**
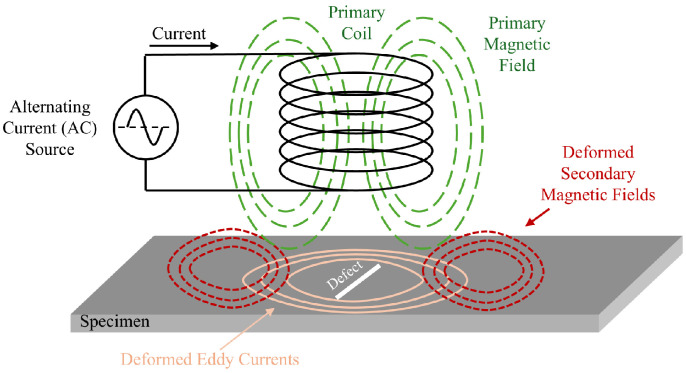
A closer and more detailed look at ECT. Photo retrieved from [108].

Remote Field Testing (RFT) is a form of eddy current testing (ECT), specifically designed for inspecting small-diameter ferromagnetic pipes (Figure 7). Although we do not explicitly explore ECT in this paper, we refer to Figure 8 for reference to the mechanism of the coils within RFT, which are further explored in this section. A typical device consists of two coils, one transmitter and one receiver, spaced out two to three pipe diameters away from each other (Figure 7). The transmitter coil emits a low-frequency (typically 50–400 Hz) alternating magnetic field radially to the pipe wall, which then induces a magnetic field in the wall, which is then picked up by the receiver coil [105,106]. Due to the nature of EC testing, RFT is limited to ferromagnetic pipes such as cast iron and steel, or concrete pipes such as PCCP, where steel wires are present within the pipe [107]. Due to the high magnetic permeability and electrical conductivity of ferromagnetic materials, electromagnetic fields exhibit strong attenuation, with skin depths on the order of millimeters or less.

Consequently, the direct field from the exciter coil is rapidly attenuated as it attempts to penetrate the tube wall. However, at distances exceeding approximately two tube diameters from the exciter coil, the so-called “remote field zone”, a fundamentally different field distribution emerges. In this remote region, the dominant magnetic field has traveled outward from the exciter coil, through the tube wall to the exterior, propagated axially along the outside of the tube, and then re-entered through the tube wall to reach a detector coil positioned inside the tube. This field path means the magnetic flux has traversed the tube wall twice: once on exiting and once on re-entering. The key phenomenon that makes RFT valuable is that in the remote field zone, the phase and amplitude of the detected signal are primarily determined by the electromagnetic properties and thickness of the tube wall rather than by the standoff distance between the detector and the inner tube surface. This characteristic contrasts sharply with conventional ECT, where lift-off variations dominate the signal response. The through-wall propagation path also provides approximately equal sensitivity to defects on either the internal or external tube surface, a significant advantage for detecting external corrosion, which is often difficult to assess from internal inspections.

A typical RFT inspection system consists of an exciter coil, one or more detector coils positioned in the remote field zone (typically 2–3 tube diameters behind the exciter), signal processing electronics, and data acquisition systems. The probe assembly is pulled or pushed through the tube at controlled speeds, continuously recording amplitude and phase information. Modern digital RFT systems employ multiple detector coils to provide redundancy, improve signal-to-noise ratios, and enable defect characterization through differential measurements. Signal interpretation in RFT focuses primarily on phase angle measurements, as phase is less sensitive to probe wobble and velocity variations than amplitude. Defects typically produce characteristic phase advances (earlier arrival of the signal) corresponding to the reduction in electromagnetic path length through the thinned wall. The magnitude of phase change correlates with the severity of wall loss, enabling quantitative assessment of remaining wall thickness.

RFT has become a standard inspection method for carbon steel heat exchangers and boiler tubes in power generation, petrochemical, and refining industries. Detection sensitivity typically allows identification of wall loss exceeding 10–20% of nominal thickness, with better performance for more extensive defects. Unlike conventional eddy current testing, RFT is minimally affected by conductive deposits on tube surfaces, ferromagnetic support plates, or tube sheets, making it particularly valuable for in-service inspection of heat exchangers without requiring extensive cleaning [109].

Despite its advantages, RFT has inherent limitations. The low operating frequencies necessary for adequate wall penetration result in relatively poor spatial resolution compared to conventional eddy current methods, making precise defect localization and sizing of small, localized anomalies challenging. The technique is also restricted to ferromagnetic tubular products and cannot inspect non-ferromagnetic materials such as stainless steel, copper alloys, or titanium. Additionally, RFT provides limited information about defect circumferential extent and cannot reliably distinguish between internal and external defects without complementary measurements. The inspection speed is typically slower than conventional eddy current testing due to the need for lower frequencies and the physical separation between exciter and detector coils.

Most RFT tools are designed for oil and natural gas pipelines, whereas fewer are designed for water and wastewater pipelines; however, some such devices exist, such as the HydraSnake. This is a tethered flexible tool designed to measure the remaining wall thickness and corrosion defects of cast and ductile iron pipe. As the tool travels through the pipe, it continuously records the wall thickness and stores the information on board. The diameter of the tool is significantly smaller than the inner diameter of the pipe, to allow for protrusions, lining, and to navigate through tees and short radius elbows [110]. The low dimensionality of the tool allows its insertion into water mains via hydrants, meaning no excavation is required.

### 4.4. Ground Penetrating Radar (GPR)

**Figure 9:**
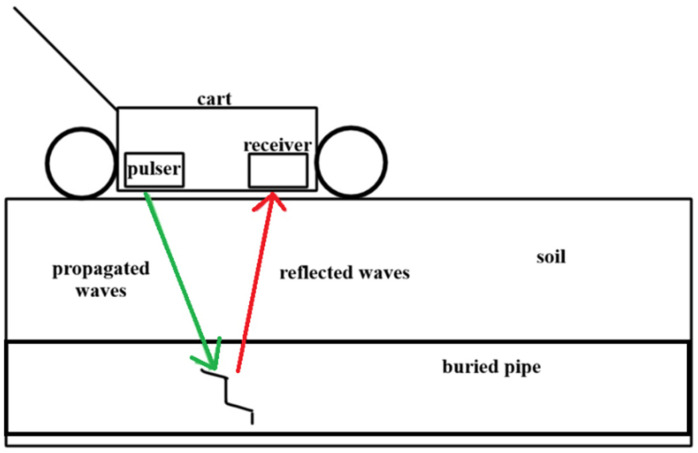
Schematic illustration of a GPR system on a wheeled cart.

Ground Penetrating Radar (GPR) uses electromagnetic wave propagation and reflection to image subsurface structures and detect buried objects. GPR operates by transmitting short pulses of electromagnetic energy, typically in the frequency range of 10 MHz to 2.5 GHz, into the ground or test material through a transmitting antenna. As these electromagnetic waves propagate through the subsurface, they encounter boundaries between materials with different dielectric properties, characterized by variations in electrical permittivity, conductivity, and magnetic permeability. At each interface where electromagnetic properties change, a portion of the incident energy is reflected back toward the surface while the remainder continues to propagate deeper into the material. A receiving antenna detects the reflected signals, and the system records both the amplitude and two-way travel time of the returning waves. The detected waves can be used to determine the geometry and depth of whatever material has reflected the radiation. Owing to a significant difference in the electromagnetic properties of water and soil, GPR can be used to locate water pipeline leaks by detecting the presence of excess saturated soil surrounding a pipe [111], which can be metallic or non-metallic, and can be in any location, e.g., rural or urban. This is often done in the field using a pulser and receiver on a wheeled cart that is pushed down the length of the buried pipe, as is done by Geophysical Survey Systems Inc [112] (Figure 9).

Recent improvements have been made to the technology in terms of better resolution, deeper surface penetration, and imaging capabilities. For example, Mizutani et al. [113] proposed a preprocessing technique based on cross-correlation to eliminate noise in GPR data. Only data points above a certain threshold correlation value are retained and then transformed into a point cloud indicating the location of things like the pipe itself or things like the location of a leak. For determining the location of a leak, particularly on the bottom of a pipe, the weak diffraction of the wet subsurface is often covered/masked by the reflections of the pipe itself. Filters to separate the diffractions are thus necessary to accurately map the leakage by eliminating particularly strong reflections which are associated with the pipe. Using 3D imaging on this isolated data provides a clear image of the position and scale of the leakage zone.

### 4.5. Microwave Testing

**Figure 10:**
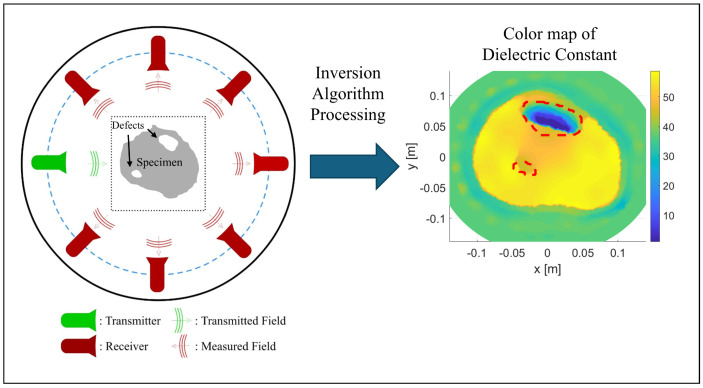
Visualization of microwave testing and model creation. Photo retrieved from [108].

The use of nonmetallic and composite pipes is on the rise, mostly because they are lightweight and not susceptible to corrosion. From an inspection standpoint, a major disadvantage is that many well-established NDE methods cannot be used [114,115]. For example, ultrasounds are highly attenuated when propagating along or through rubber or soft plastics [114]. Electromagnetic methods, such as MFL, MMM, and RFT, are only viable options for ferromagnetic pipes, and radiography methods cannot identify defects caused by the separation of internal component layers, leading to a large blind spot in those results. However, microwave testing can be used to develop volumetric models of the cylindrical composite pipes such that these models may be examined for wall thickness, presence of faults, and delamination or internal separations. Microwave testing apparatus for a water pipe consists of a reflectometer connected to a movable antenna. The antenna radiates a continuous microwave beam into the pipe from different directions. The wave is backscattered as it passes through discontinuities [114,115]. By scanning at predetermined grid points along the pipe, the 2D scan can be mapped to a 3D model, allowing the detection and location of flaws, as schematized in Figure 10.

Microwave testing is similar to ultrasonic testing without the need for physical contact or the use of a couplant [114]. Evisive used the methodology to evaluate high-density polyethylene (HDPE) thermal butt fusion welds [116]. Compass Technology Group Inc. utilizes microwaves sized 2–18 GHz or 20–40 GHz with their microwave calipers that allow microwave testing to be performed in situ [117].

### 4.6. Fiber Optics

**Figure 11:**
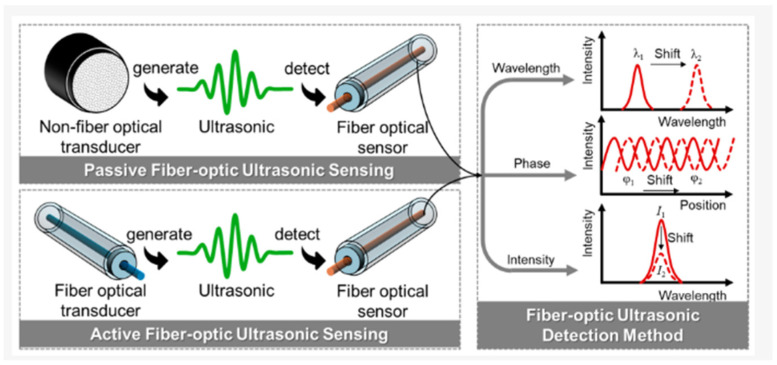
Passive vs. active fiber-optic sensing. Photo retrieved from [118].

With the increase in research and development of SHM technologies, fiber optic technologies are being considered for pipe monitoring. The methodology uses light-to-sound conversion mechanisms to detect stress waves (ultrasounds) and convert them into a light beam to be propagated along an optical fiber, which does not suffer from electromagnetic interference. Established techniques include Brillouin, Raman, and Rayleigh scattering-based methods [118]. This ultrasonic detection can be done passively or actively, by either exciting the fiber by generating ultrasonic waves externally (via piezoelectric transducers or acoustic emissions) or with a fiber-based transducer (Figure 11). Either way, the fiber optic sensors detect the perturbations in their optical signal and are able to then demodulate these changes and produce graphs that visualize the waveform for analysis [118]. Although this methodology uses ultrasonic waves, the core tenet is the traveling of light/optic energy through the optical fiber, preserving fiber optics’ distinction as an electromagnetic method [118,119].

Advantages of fiber optics include its light weight, small size, real-time results, and immunity to electrical noise and electromagnetic interference [119]. Disadvantages include the complexity of the results, which require specialized training to interpret, the loss of signal integrity over long distances, and little to no information about the accuracy of fiber optic sensors in rugged environments such as those with high radiation and/or high humidity. APSensing offers Distributed Fiber Optic Sensing (DFOS) technologies for the continuous monitoring of pipelines, both buried and above-ground, for leaks, ground movement, and liquid accumulations affecting flow [120]. According to APSensing, their DTS N45-Series offers up to 70 km of range and stability after years of monitoring a harsh environment [121]. The DTSS N62-Series of distributed temperature and strain sensing technology offers over 80 km of range and minimal maintenance [122]. Both series can be linked to APSensing’s SmartVision software [123].

## 5. Probabilistic Methods

As is said in the Introduction, traditional NDE-based inspections are either performed on a periodic basis or are performed reactively, i.e., after a fault has occurred and an incident such as a flood or blockage has been reported [48]. Because pipe rehabilitation or replacement is expensive, chronically underfunded, and causes service disruptions, condition assessment methods must be balanced with cost analyses to optimize available budgets. Rehabilitating a water main prematurely is economically suboptimal, while delaying intervention until failure leads to catastrophic consequences and emergency repair costs. Consequently, research and development and owners’ practice have focused on analytical techniques that can identify optimal timelines for repair, replacement, and rehabilitation of pipe networks [124]. This need is increasingly recognized, as “many drinking water utilities are actively improving infrastructure through innovations such as asset failure prediction technologies, which improve the ability to identify issues before they become failures” [5]. These proactive measures are usually implemented using probabilistic modeling and failure-cost analysis.

One approach to performing failure-cost analysis involves developing Life Assessment Models (LAMs), which use economic factors as the primary driver for determining when to repair or replace infrastructure. LAMs model and simulate the deterioration of buried water and wastewater pipes by accounting for operational costs, repair costs, failure expenses, and depreciation charges on an annual basis. Under this framework, a pipe requires replacement or rehabilitation when the cost of maintaining its current condition meets or exceeds the cost of intervention or new asset installation. Key pipe parameters incorporated into LAMs include diameter, material, age, length, and reliability [125]. LAMs perform optimally when applied to newer pipes with well-documented installation histories, usage patterns, and material specifications. For older infrastructure, however, accurate modeling becomes challenging due to incomplete or uncertain information, potentially yielding less reliable predictions. Nevertheless, LAMs are designed as continuously iterative systems: the longer they operate while receiving operational data and making predictions, the more accurate they become. This characteristic encourages implementation even for pipe networks with high initial uncertainty [125].

Zangenehmadar, Moselhi, and Golnaraghi [126] advanced this concept by developing an optimization model using Montreal’s water distribution network. Their approach seeks to identify near-optimal maintenance schedules for networks containing *n* individual pipes at year *t*, with a planning horizon of *T* years (*T* = *t* − 2018) and a limited annual budget *B_t_* for maintenance and repair. The model predicts *K_i,t_* (*i* ∈ [1, *n*]), which is the number of leaks for pipe *i* in year *t* that will result in breaks if no intervention occurs. By integrating the number of pipes, planning horizon, budget constraints, and projected leak frequencies as inputs, the model generates cost-based rehabilitation plans that prioritize the most urgent repairs while remaining within budget limitations.

Another probabilistic methodology combines Monte Carlo simulation with fuzzy logic to address uncertainty in pipe condition assessment. This approach uses pipe characteristics—including diameter, thickness, age, material, water pressure, joint types, maintenance practices, leakage history, and failure records—as inputs to a Monte Carlo Simulation Model to calculate a deterioration index for the water network. These outputs are then processed through a Fuzzy Inference System (FIS) to account for uncertainty and incompleteness in the input data. The FIS generates a failure index ranging from 0 to 100, where 100 indicates no risk of failure, and 0 represents certain failure. This failure index can then inform budget optimization and strategic planning for water distribution networks [124].

Probabilistic risk-based modeling has been implemented commercially through the Ductile Iron Pipe Research Association and Corrpro’s Design Decision Model (DDM). This model focuses specifically on corrosion control by evaluating two key factors: the corrosivity of the soil environment in which pipes are buried (Likelihood) and the magnitude of consequences resulting from potential failures (Consequence). By analyzing these factors, the DDM recommends optimal protective coatings to mitigate corrosion. While simpler than the academic models discussed above, the DDM represents a practical implementation of probabilistic methodology for optimizing cost-effectiveness and service life extension in water pipe infrastructure [127].

An emerging concept in probabilistic SHM is population-based modeling, which addresses the challenge of data scarcity in aging infrastructure. This approach, though not yet practically implemented, proposes a transfer learning framework wherein data from well-instrumented, data-rich structures that have been subjected to various damage scenarios can be applied to similar but data-poor structures [2]. The methodology leverages observations and measurements from a population of accessible structures to make inferences about individual structures characterized by high uncertainty. This approach directly addresses two of the most significant obstacles in probabilistic methods: the uncertainty inherent in aged systems and the difficulty of obtaining real-world operational data for model training. In future studies, using data-rich structures in conjunction with unsupervised machine learning and deep learning methods may prove to be beneficial in obtaining the location of failures and identifying the most vulnerable sections of a structure. Wang and Cha [128] have shown that such methods have had success on a small scale.

Despite the promise, probabilistic methods face several significant challenges. The most prominent obstacle is the high degree of uncertainty associated with aging infrastructure, particularly regarding historical installation practices, material properties, operating conditions, and maintenance histories. Additionally, the scarcity of real-world failure data and operational measurements limits the ability to train and validate predictive models effectively. For older pipe networks with incomplete documentation, model accuracy is inherently constrained, though iterative refinement can improve predictions over time as operational data accumulates. These limitations underscore the need for hybrid approaches that combine probabilistic modeling with other NDE and SHM techniques to provide comprehensive condition assessment capabilities.

## 6. Discussion and Conclusions

This comprehensive review has examined the principal NDE and SHM techniques currently employed for water and wastewater pipeline inspection, discussing significant technological progress and implementation challenges. The review was categorized into visual approaches, mechanical wave-based techniques, electromagnetic methods, and probabilistic frameworks. A critical observation emerging from this review is the disparity in research and technological development between water/wastewater infrastructure and oil and gas transmission systems. This gap stems primarily from economic considerations and secondarily from technical barriers. The lower commodity value of water compared to petroleum products, combined with lower environmental remediation costs associated with water leaks, has historically resulted in diminished priority for NDE technology advancement in the water sector. In addition, the fact that most pipelines are buried underground and cannot be subjected to any form of contamination poses technical constraints that limit the number of potential NDE solutions.

Despite this disparity, recent years have witnessed substantial improvements in water pipeline NDE capabilities. These advancements largely derive from the integration of emerging computational technologies, AI (which includes machine learning algorithms), IoT connectivity, and robotics, that enhance detection sensitivity, reduce inspection time, minimize personnel requirements, and improve overall cost-effectiveness.

Visual Inspection retains its position as the most widely deployed technique, particularly for wastewater systems and targeted investigation of suspected leak locations. The evolution from basic CCTV systems to sophisticated panoramic imaging platforms capable of generating three-dimensional pipe models represents substantial progress. Modern systems achieve approximately 90% defect detection accuracy while maintaining relatively low operator skill requirements and equipment costs. However, the fundamental limitation remains: visual methods require internal pipe access, flow interruption or significant reduction, and manual review for defect identification despite computational assistance. These constraints restrict visual inspection primarily to reactive assessment rather than proactive monitoring applications.

Mechanical wave-based techniques demonstrate versatility in deployment configurations, supporting both periodic inspection implementation and continuous monitoring installations. The diversity of transduction mechanisms, such as accelerometers, hydrophones, and piezoelectric transducers, as well as configurations (active/passive, pitch-catch, pulse-echo, guided wave), enables customization to specific operational requirements. ALD systems, particularly when integrated into SWN architecture, provide continuous monitoring capability for defined geographic areas, typically urban zones where leak costs and social impacts are greatest. These systems exploit low-frequency structural vibrations or low-frequency sound propagating through water to identify and localize leakage events.

AE monitoring, widely successful in bridge and pressure vessel applications to name a few, offers passive detection of crack initiation and propagation through monitoring stress wave emissions within the pipe material itself. While providing early warning of developing failures, AE effectiveness is limited by shorter monitoring ranges compared to ALD, as stress waves attenuate more rapidly in pipe walls than acoustic signals in water. Both ALD and AE face significant challenges from ambient noise, particularly prevalent in urban environments where vehicular traffic, machinery, and other anthropogenic sources mask leak signatures.

UT maintains its status as the most established NDE method globally, benefiting from extensive commercial development, diverse implementation options, and substantial operator experience. The distinction between bulk waves and GUW approaches reflects fundamentally different inspection philosophies: bulk waves provide localized, high-resolution through-wall characterization requiring systematic scanning but offering superior defect characterization; GUW techniques enable long-range screening from single test locations at the expense of spatial resolution and defect sizing precision. Recent innovations, such as the Synthetic Aperture Focusing Technique (SAFT), enhance both approaches through advanced signal processing that effectively expands inspection coverage and improves defect visualization.

EM for water pipe inspection was categorized into MFL, MMM, RFT, and GPR, each addressing distinct inspection scenarios. MFL, despite its widespread adoption in natural gas pipeline inspection through inline inspection vehicles (PIGs), faces implementation barriers in water applications, including contamination concerns, dry pipe requirements, restrictions on ferromagnetic materials, and substantial capital costs. Recent neural network integration for automated defect recognition has improved MFL practicality, yet material constraints fundamentally limit applicability across the diverse pipe material spectrum characterizing water infrastructure.

GPR provides complementary capabilities through above-ground deployment, eliminating internal access requirements while offering three-dimensional subsurface imaging for leak zone localization in buried pipelines. Technological improvements, particularly in spatial resolution and data visualization, have enhanced GPR effectiveness for water infrastructure assessment, though interpretation expertise and site-specific electromagnetic property variations influence reliability. Microwave testing is ideal for nonmagnetic pipelines, such as those made of composites, allowing for accurate analysis of non-metal pipes, which are rising in popularity.

Fiber optic sensing is expanding quickly and shows much promise for its integration into pipelines for continuous and proactive monitoring, with newer technologies increasing the range and building the sensors for harsher environments.

Probabilistic Methods offer strategic-level decision support rather than defect detection capabilities. Life assessment models (LAM) employ statistical frameworks to optimize replacement and rehabilitation scheduling by comparing maintenance costs against renewal expenses, triggering intervention when the former equals or exceeds the latter. Model accuracy depends critically on detailed pipe characteristic data—diameter, material composition, installation date, operational history, soil conditions, and consequence severity—information frequently unavailable for aged infrastructure installed prior to comprehensive record-keeping practices. Various probabilistic modeling approaches have been proposed, incorporating pipe-specific parameters, environmental factors (soil properties, groundwater chemistry), and failure-consequence magnitude. Population-based SHM models, which infer individual structure condition from population statistics, hold theoretical promise but face practical implementation obstacles due to insufficient real-world training data and incomplete legacy system documentation. Nevertheless, when integrated with direct NDE measurements, probabilistic methods provide valuable strategic planning tools for prioritizing inspection resources and optimizing capital investment allocation across extensive distribution networks. We summarize these comments in Table 1 with a focus on this review’s prioritized characteristics. A couple of notes to add for Table 1: we define proactive use as the ability to find a flaw before leakage occurs, and automation is based on the amount of human labor put into detecting and flagging defects from setup to decision making. For example, visual methods may catch a leak before it turns into a blowout; we place it into the reactive category as it is not able to identify a forming crack. We additionally say visual methods are only partially able to be automated, as manpower is required to collect the footage that is to be evaluated via automation.

Overall, the ongoing integration of AI, including deep learning architectures for automated defect recognition and classification, promises to address persistent limitations, including interpretation subjectivity, labor intensity, and inspection speed constraints. Similarly, IoT connectivity enables real-time data aggregation from distributed sensor networks, supporting rapid response to developing failures and data-driven optimization of inspection intervals. Several key conclusions can be drawn from this review:

1. Substantial progress in water pipeline NDE derives largely from adaptation of techniques developed for oil and gas applications, reflecting economic rather than technical limitations. However, the critical importance of water and wastewater infrastructure to public health, economic productivity, and environmental sustainability demands continued innovation in NDE/SHM capabilities.
2. No universal solution exists.
3. The maturity of certain technologies and the market dominance of some private industries suggest focusing on the improvement of software and hardware, rather than the investigation of new solutions. Continued advancement will likely emphasize automation through machine learning, distributed sensing through IoT networks, and decision integration through comprehensive asset management frameworks.
4. Water infrastructure presents unique challenges compared to hydrocarbon systems, including service continuity requirements, material heterogeneity, accessibility limitations, contamination, and constrained budgets.

Successful NDE implementation must accommodate these realities.

## Figures and Tables

**Table 1:** Overview of all methods.

Method	Enable Automation?	Main Limitations
Visual	Partially	Require internal access and sufficient lighting, with only surface defects detectable. Requires defects to already be visible. Cannot see through slime or dirt (requires surface preparation).
AE	Yes	Sensitive to environmental noise. Requires pressure and flow
ALD	Yes	Requires existing leaks for detection. Sensitive to environmental noise.
ACIE	No	Requires an existing leak for soil void detection. Sensitive to environmental noise.
Bulk UT	Yes	Requires surface preparation (couplant).
GUW	No	May miss defects parallel to waves.
Fiber Optic	Yes	Installation cost. Fibers’ brittleness.
MFL	Yes	Ferromagnetic materials only. Detects existing defects
MMM	Yes	Ferromagnetic materials only. Yields only qualitative results. Sensitive to external magnetic fields
RFT	Yes	Ferromagnetic materials only. Time consuming
GPR	Partially	Soil dependent.
Microwave	Yes	Unable to be used on ferromagnetic materials. Low penetration.
Probabilistic	Yes	Accuracy is dependent on software and training data quality.

## Data Availability

There is none to be made available.
